# Physical Education in a Thermal Spa Resort to Maintain an Active Lifestyle at Home: A One-Year Self-Controlled Follow-Up Pilot Study

**DOI:** 10.1155/2017/1058419

**Published:** 2017-04-30

**Authors:** Julien Maitre, Benjamin Guinhouya, Nicole Darrieutort, Thierry Paillard

**Affiliations:** ^1^Laboratoire Mouvement, Equilibre, Performance et Santé, EA 4445, Université de Pau et des Pays de l'Adour, Département STAPS, 11 rue Morane Saulnier, 65000 Tarbes, France; ^2^EA 2694, Santé Publique: Épidémiologie et Qualité des soins et UFR Ingénierie et Management de la Santé, Université de Lille, 59000 Lille, France; ^3^Medical Office, 6 rue Larrey, 65200 Bagnères-de-Bigorre, France

## Abstract

The self-controlled follow-up pilot study was set up to examine the maintenance of engagement in physical activity by a group of older adults in a thermal spa resort, as a consequence of the inclusion of additional physical education sessions within their usual care offers. A cohort of 42 participants (70.4 ± 4.5 years) underwent three weeks of thermal treatment with additional physical education (PE) sessions. Measurements were established during the intervention in 2 periods (baseline and final thermal treatment evaluation) and 4 periods of measurements in the follow-up (+15 days, +2 months, +6 months, and +1 year). Physical measures (anthropometrics, flexibility, and 6-minute walk test) and intrapersonal and psychosocial factors as well as health-related quality of life (HQOL) and physical activity (PA) were self-reported by participants. Only HQOL and PA were assessed during the follow-up. One year after a 3-week PE session combined with the usual thermal care, 64% of the participants exhibited a higher volume of PA than at baseline. The components of the HQOL changed during the follow-up. This strategy to maintain PA engagement appears to be feasible in a population of thermal care older adults. This work demonstrates the feasibility of a study conducted to maintain physical activity engagement after a thermal treatment.

## 1. Introduction

Balneotherapy and spa therapy are essential treatments in thermal spa resorts and have been widely used for health promotion, prevention, treatment, and rehabilitation in European countries [1–3]. Previous studies have suggested not only a symptomatic improvement but also an improvement in the psychological condition and quality of life of participants [4, 5].

In thermal spa resorts the care of older adults, which usually spans a period of 3 weeks [6], not only allows them to benefit from treatments but also allows them to receive health education and/or to undergo health promotion actions on specific issues associated with their condition. In this respect, physical activity could be viewed as an added-value strategy to reinforce the therapeutic effects of the ongoing treatment.

To reinforce the effects of therapeutic treatments, physical activity could be added to thermal treatment for the beneficial effects of regular exercise [7]. Indeed, the preventive effects of regular physical activity are clearly evident at the metabolic, osteoarticular, muscular, neurological, and psychological levels [8]. Yet, to the best of our knowledge, physical activity is rarely added to the thermal treatment. The three weeks usually prescribed for a thermal treatment could be an excellent opportunity to include physical education sessions within healthcare. Although the effects of regular physical activity [8, 9] and thermal treatment contribute to improving health, there are still too few studies that focus on the effects of exercise during a thermal treatment period [4, 10, 11]. In addition, no study has thoroughly examined the role that additional physical education sessions may play during a period of thermal treatment in thermal spa resorts on the maintenance of engagement in physical activity after this intervention period. This is a new approach in the field of thermal care that is still little addressed and deserves to be analysed, through a prospective interventional study. Hence, the purpose of this pilot study was to examine the maintenance of engagement in physical activity by a group of older adults in a thermal spa resort as a consequence of the inclusion of additional physical education sessions within their habitual care. Indeed, improvements of physical fitness and psychological status are related to both adoption and maintenance of physical activity practice [12–14]. We hypothesized that, after a physically active period of thermal treatment, a group of participants would continue to perform their acquired level of physical activity after their return home.

## 2. Methods

### 2.1. Participants and Study Design

A cohort of 42 participants (mean age: 70.4 ± 4.5 yr, height: 161.8 ± 8.0 cm, and weight: 74.6 ± 14.21 kg), 33 women and nine men, voluntarily participated in this study and gave their written informed consent. The experiments received the approval of the local committee for the protection of human participants. All the participants were recruited in the same French thermal spa resort at the beginning of their 21-day thermal treatment.

Participants were included after a medical examination. Inclusion criteria were (1) to be 65 years old or more, (2) to present at least one of the listed pathologies (i.e., rheumatological diseases, respiratory diseases, otorhinolaryngological diseases, or fibromyalgia), and (3) to be considered as low active according to the test of De Souto Barreto et al. [15]. Exclusion criteria were (1) to be involved in any exercise in a sports club, (2) to present a history of major disorders (e.g., muscular, neurological, metabolic, and cardiovascular) that would limit their participation in exercise, (3) to be under medication that could alter their heart rate, (4) to present cardiovascular, neurological, or respiratory (i.e., requiring treatment with oxygen therapy) disorders, (5) to receive medical treatment (i.e., beta-blockers, corticosteroids, and neuroleptics), and (6) to present a traumatism (in the past 2 years) or ankylosis of a large lower limb joint (hip, knee, and ankle).

#### 2.1.1. Procedure

**Figure 1:**
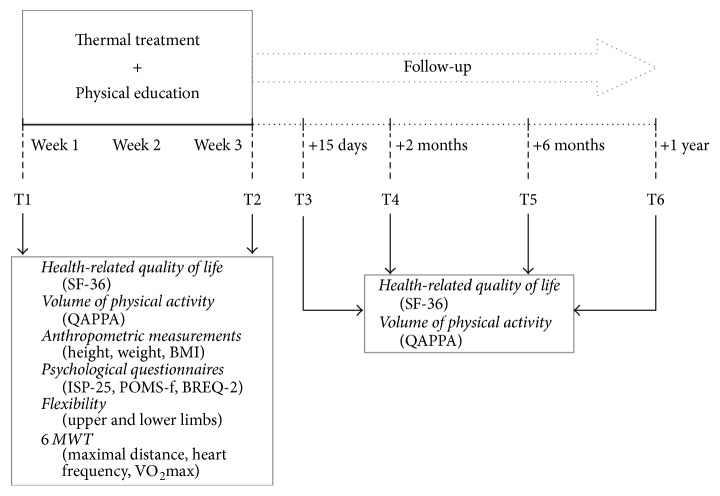
Procedure of the experimental protocol.* Note*. T1, baseline evaluation; T2, final thermal treatment evaluation; T3, T4, T5, and T6 correspond to follow-up at 15 days, 2 months, 6 months, and 12 months after the end of the thermal cure (5 to 7 treatments per day) + physical education sessions (4 per week).

Over a period of 21 days, the thermal treatment provided to participants consists in 5 to 7 health treatments per day (i.e., thermal water baths, thermal water showers, thermal mud baths, thermal water inhalations, and massages). Together with this standard care, adapted physical education sessions were provided to them for three weeks (i.e., 12 sessions, four per week). Measurements were established on 6 periods. During the intervention, 2 measurement periods were established (T1 and T2) and 4 measurement periods during the follow-up (T3, T4, T5, and T6) (Figure 1).

Measurements of anthropometric characteristics, physical fitness, psychological parameters, and health-related quality of life (HQOL) were conducted at the baseline evaluation (T1) in participants. The final thermal treatment evaluation (T2) included the evaluation of anthropometric characteristics, physical fitness, psychological parameters, and HQOL at the end of the thermal treatment (i.e., after healthcare and physical education programs). The follow-up consisted in testing sessions held 15 days (T3), 2 months (T4), 6 months (T5), and 1 year (T6) after the end of the thermal treatment. During the follow-up, only measurements of physical fitness and HQOL were conducted (Figure 1).

### 2.2. Anthropometric Measurements

Height and weight were assessed, respectively, with a height gauge (i.e., heels touching each other, upright posture) and a weight scale (i.e., without shoes) by the same investigator for each measure (Figure 1). The body mass index (BMI) was calculated at each time measurement.

### 2.3. Physical Activity and Physical Fitness Measurements

#### 2.3.1. Physical Activity

The volume of physical activity was assessed through a French self-reported physical activity questionnaire, the “Questionnaire d'Activité Physique pour les Personnes Agées” (QAPPA) [15]. QAPPA had acceptable test-retest reliability (*r* = 0.58, *p* < 0.001) and good reproducibility (*k* = 0.714). This questionnaire was used to assess the volume of physical activity (MET-min/week). The data from the self-reported volume of physical activity were assessed at each time measurement (Figure 1).

#### 2.3.2. Maximal Oxygen Uptake (VO_2_max)

Participants were instructed to walk, alone, the maximal distance they could during a 6-minute period—that is, the 6-minute walk test (6 MWT)—under the supervision of the investigator. This test was performed at T1 and T2. Participants wore a Polar™ RS400 monitor (Polar Electro, Kempele, Finland) to record their heart rate (HR).

VO_2_max was estimated during the 6 MWT according to a predictive equation [16]. For calculating VO_2_max the HR was averaged from the last two minutes.

(1) VO2maxL/min=−1.732+body weightkg×0,049+distancem×0.005+HRbeats/min×−0.015.

#### 2.3.3. Flexibility of the Upper and Lower Limbs

To assess the flexibility of the trunk and the lower limbs, participants were assessed alone by the investigator. They were placed in a sitting position, legs straight, back straight, and arms outstretched in front of them. A flexometer was used to measure the distance (i.e., range of −20 to +35 cm) from the tip of the fingers to the toes (hands juxtaposed) achieved by the participants. To assess the flexibility of the upper limbs participants were placed with their back to a bar (e.g., a handrail), legs straight, and their hands grasping the bar (between the thumb and the fingers) and their arms were outstretched. The participants were instructed to lower their pelvis by flexing their legs and to move their hands as close as possible to each other while keeping their arms outstretched. The degree of flexibility was determined by measuring the distance remaining between the two hands on the bar. The flexibility data were assessed at T1 and T2 (Figure 1).

### 2.4. Psychological Factors

Participants were asked a set of psychological questions at baseline (T1) and then again at the end of the thermal treatment (T2). The Physical Self-Perception Profile (ISP-25, French version), the Behavioural Regulation in Exercise Questionnaire (BREQ-2, French version), and the Profile of Mood States (POMS-f, French version) were used for data collection. The ISP-25 was used for the assessment of the global self-esteem in the physical domain [17] covering 6 factors (global self-esteem, physical self-worth, endurance, sport competence, attractive body, and physical strength). The BREQ-2 was used to measure external, introjected, identified, and intrinsic forms of regulation of exercise behaviour and amotivation [18]. The POMS-f was used to measure 7 factors (anxiety, depression, anger, confusion, fatigue, vigour, and friendliness) and determine a global score [19].

### 2.5. Health-Related Quality of Life

To assess the health status of participants, the French version of the Short Form-36 (SF-36) questionnaire was used [20]. Participants were asked to complete this questionnaire at each time measurement (Figure 1). The SF-36 contains 9 scales for assessing physical functioning (PF), role limitations relating to physical health (RP), bodily pain (BP), general health perceptions (GH), vitality (VT), social functioning (SF), role limitations relating to mental health (RE), mental health (MH), and health transition (HT).

### 2.6. Physical Education Sessions

After the baseline evaluation (T1) all the participants underwent an adapted physical activity program that had been individually tailored (i.e., type of exercise, duration, and intensity) to enhance the interest, motivation, and engagement in physical activity of each participant. The duration and intensity of the physical education sessions were continuously adjusted according to the participants' feelings (i.e., stereotyped questions) and with objective values (i.e., training load). The sessions were planned over a period of three weeks at the rate of four sessions per week. Participants were asked to stay at rest on Wednesday and weekends. The sessions were completed in groups of three participants maximum (there were 432 individual sessions, 30 sessions per group of two participants and 4 sessions per group of three participants).

One of the main goals of these additional physical education sessions was also to increase physical fitness (i.e., aerobic fitness and force abilities), in order to improve the efficiency of postural control and coordination, and to increase the flexibility of participants. These enhancements were achieved through 2 types of physical education sessions. Each type of session was alternated and lasted one hour (warm-up, training exercises, and cooling down). There were brisk walking sessions (e.g., warm-up: 10 minutes of slow walking; training exercises: 40 minutes of brisk walking; cooling down: 10 minutes of stretching) and gymnastic maintenance sessions (e.g., warm-up: 15 minutes of joint mobilisation; training exercises: 10 minutes of balance exercises, 10 minutes of coordination exercises, and 15 minutes of muscular strengthening; cooling down: 10 minutes of stretching).

### 2.7. Statistical Analysis

The normal distribution of continuous data was checked using a Shapiro-Wilk test. Because normality was skewed in most cases, the data on age, BMI, flexibility (i.e., upper and lower limbs), 6 MWT (i.e., heart rate, maximal distance, and VO_2_max), ISP-25, BREQ-2, and POMS-f obtained before (T1) and after (T2) the thermal treatment (+ physical education sessions) were compared using a rank order Wilcoxon test.

A Friedman test was performed to determine whether there were differences between T1, T2, T3, T4, T5, and T6 in the HQOL components or in the volume of physical activity. When a significant treatment effect occurred, a Wilcoxon test with a Bonferroni correction to type I error was used to test any pairwise difference.

Finally, a multilevel modelling was performed on the normalised data (log-transformed) of physical activity volume to examine its course over six periods of measurement. The crude model was further adjusted in the HQOL components, which were found to be significantly related to physical activity (i.e., by the use of Spearman's rank correlation coefficient).

Statistical treatment of data was achieved using the SPSSv19 software (IBM SPSS statistics, USA). Results were considered significant at the level of 5%.

## 3. Results

### 3.1. Anthropometric Characteristics and Physical Fitness

As shown in Table 1, the BMI of participants was significantly lower in T2 than at the baseline. Flexibility (i.e., upper and lower limbs) was significantly greater in T2 than in T1 (Table 1). During the 6 MWT, maximal walking distance, heart rate of the last two minutes of the test, and VO_2_max significantly increased from baseline to the end of the thermal cure (Table 1).

### 3.2. Psychological Factors

Some factors of the ISP-25, BREQ-2, and POMS-f presented a significant change from baseline to the end of the thermal cure (Table 2). Concerning the ISP-25, global self-esteem, and physical self-worth presented a significant increase. With regard to the BREQ-2, intrinsic regulation showed a significant increase from baseline to the end of the thermal cure. In addition, identified regulation presented a tendency (*p* = 0.057). With respect to the POMS-f, only the friendliness factor did not present a significant change from baseline to the end of the thermal treatment. The tension/anxiety, anger/hostility, confusion/bewilderment, depression/dejection, fatigue/inertia, vigour/activity factors, and the global score significantly evolved from baseline to the end of the thermal cure.

### 3.3. Health-Related Quality of Life Components

**Figure 2:**
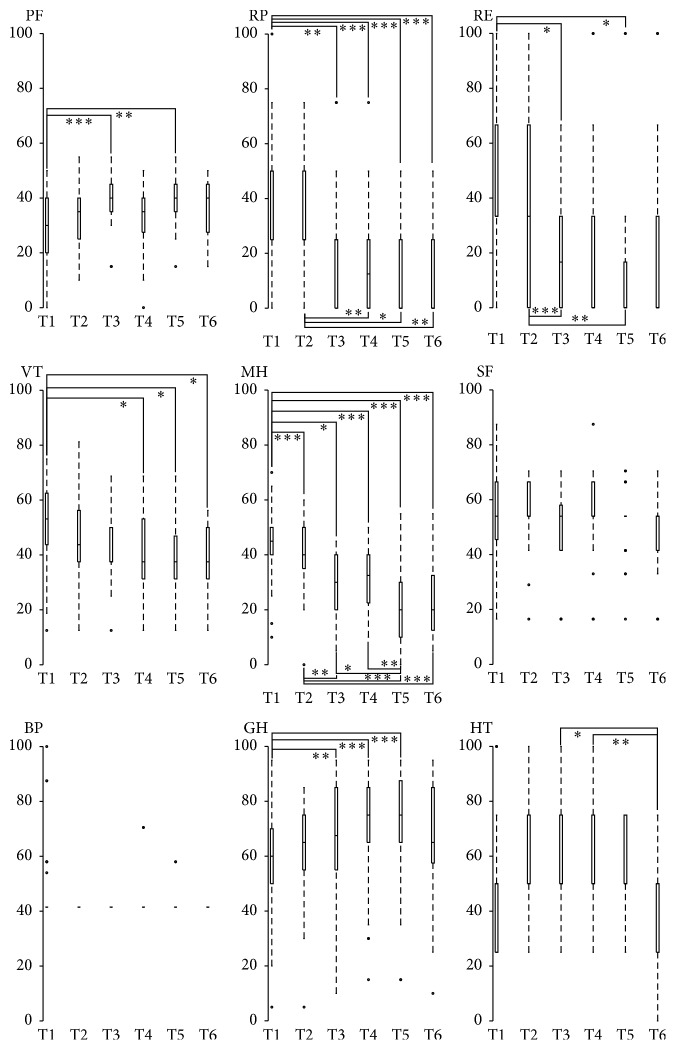
Health-related quality of life components from the SF-36 questionnaire. Data are presented in median (minimum and maximum).* Note*. T1, baseline evaluation; T2, final thermal treatment evaluation; T3, T4, T5, and T6, respectively, correspond to follow-up at 15 days, 2 months, 6 months, and 12 months after the end of the thermal treatment + physical education sessions. PF, physical function [*χ*^2^(5) = 21.90, *p* = 0.001]; RP, physical role [*χ*^2^(5) = 55.64, *p* = 0.000]; RE, emotional role [*χ*^2^(5) = 43.69, *p* = 0.000]; VT, vitality [*χ*^2^(5) = 14.10, *p* = 0.015]; MH, mental health [*χ*^2^(5) = 54.84, *p* = 0.000]; SF, social function [*χ*^2^(5) = 6.23, *p* = 0.284]; BP, bodily pain [*χ*^2^(5) = 18.15, *p* = 0.003]; GH, general health [*χ*^2^(5) = 44.31, *p* = 0.000]; HT, health transition [*χ*^2^(5) = 23.84, *p* = 0.000]. The median significance differences are included in the table at the level of 5%. Post hoc analyses correspond to Wilcoxon pairwise comparisons with Bonferroni correction: ^*∗*^*p* < 0.0033, ^*∗∗*^*p* < 0.00066, and ^*∗∗∗*^*p* < 0.000066.  ° indicates outliers.

Physical function was significantly higher at T3 and T5 than at T1 (Figure 2). The physical role was significantly lower at T3, T4, T5, and T6 than at T1. In addition, the physical role was significantly lower at T4, T5, and T6 than at T2. Concerning the emotional role, this parameter was significantly lower at T3 and T5 than at T1 and T2. Vitality was significantly lower at T4, T5, and T6 than at T1. Mental Health was significantly lower at T3, T4, T5, and T6 than at T1. Moreover, Mental Health was significantly lower at T3, T5, and T6 than at T2 and significantly lower at T5 than at T3 and T4. General Health was significantly higher at T3, T4, and T5 than at T1. Health transition at T1 was significantly lower than at T2; health transition at T6 was significantly lower than at T3 and T4.

### 3.4. Volume of Physical Activity

**Figure 3:**
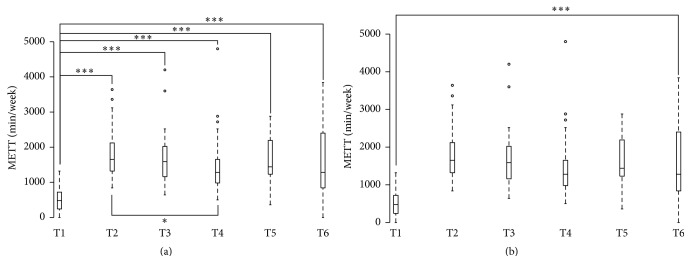
Volume of physical activity from baseline (T1) to the end of the period of thermal treatment + adapted physical education sessions (T2) and follow-up at 15 days (T3), 2 months (T4), 6 months (T5), and one year (T6).* Note*. T1, baseline evaluation; T2, final thermal treatment evaluation; T3, T4, T5, and T6 correspond to follow-up at 15 days, 2 months, 6 months, and 12 months after the end of the thermal cure + physical education sessions. (a) The nonparametric Friedman analysis of variance presented a significant main effect [*χ*^2^(5) = 59.76, *p* = 0.000]. Post hoc analyses correspond to Wilcoxon pairwise comparisons with Bonferroni correction. ^*∗*^*p* < 0.0033 and ^*∗∗∗*^*p* < 0.000066. ° indicates outliers. (b) Multilevel modelling using normalised data, with an adjustment on health-related quality of life.

The volume of physical activity was significantly higher at T2, T3, T4, T5, and T6 than at T1 (Figure 3). In addition, the volume of physical activity at T4 was significantly lower than at T2. When the HQOL components were adjusted for, there was a difference only between T1 and T6.

By taking the baseline volume of physical activity as a reference, at T2, 100% (42/42), 97.6% (41/42), 78.6% (33/42), 76.2% (32/42), and 64.3% (27/42) of the participants were more physically active at T2, T3, T4, T5, and T6, respectively, than at T1.

## 4. Discussion

The aim of this pilot study was to determine whether, after 3 weeks of a physically active thermal treatment, participants manage to maintain their involvement in an active lifestyle. The main results of this study indicated that the participants' volume of physical activity was significantly higher at 2 weeks, 2 months, 6 months, and 1 year after the end of their active thermal treatment than their baseline level. In fact, 1 year after the end of the thermal healthcare, 64% of the participants still had a higher volume of physical activity than at baseline. In addition, there was a change to the health-related quality of life components during the follow-up. Furthermore, the participants improved their physical fitness components (i.e., BMI, flexibility, 6 MWT distance, and VO_2_max) and their psychological parameters (i.e., global self-esteem, physical self-worth, intrinsic motivation, and mood states) at the final thermal treatment evaluation as compared to baseline data.

As suggested by previous studies [7, 21, 22], interventions conducted to promote regular physical activity can positively impact physical activity behaviour and other health parameters during the follow-up period (i.e., after the end of the thermal healthcare). In the present study, there was an increase in the volume of physical activity at each time point as compared to baseline. At the end of the follow-up, a high proportion (64%) of participants were engaged in more physical activity than at the baseline, which suggests that they may have benefited from the physical education sessions added to the usual thermal treatment. Indeed, at the final thermal treatment evaluation participants demonstrated better values in their ability to move than at the baseline evaluation, that is, 6 MWT, VO_2_max, and flexibility. They also improved their psychological status, that is, global self-esteem and physical self-worth, anxiety, and mood states. In addition, participants increased the values of intrinsic regulation of exercise behaviour at the final thermal treatment evaluation compared to the baseline evaluation. Intrinsic motivation, defined as the doing of an activity for its inherent satisfactions rather than for some separable consequence [23], appears to be fundamental to exercise adherence [24]. Moreover, participants demonstrate high values for identified regulation, which refers to being motivated to perform a behaviour because it is personally significant and results in outcomes which are valued by the individual [25, 26], at the baseline and the final thermal treatment evaluation. Identified regulations represent an autonomous form of extrinsic motivation [25], and extrinsic motivation (e.g., improved health) may often contribute to intrinsic interest in exercise and encourage long-term exercise adherence [24]. It is likely that the physical and psychological status of participants at the end of the intervention were better and may support their engagement in an active lifestyle.

Similarly to the volume of physical activity reported, the HQOL components changed during the follow-up (e.g., general health, physical function, role physical, role emotional, and mental health). As suggested by previous studies [27, 28], the effects of the intervention and the maintenance of an adequate volume of physical activity by most of the participants might have enabled them to perceive an improved general health and physical function. Moreover, participants expressed more difficulties in their physical role during the follow-up. Participants increased their ability to interact with the environment [29], enabling them to confront their limit more regularly than before the intervention. Elsewhere, intriguing results showed that vitality, emotional role, and mental health decreased during the follow-up. Physical activity may increase energy expenditure but may also increase the sedentary time in individuals who were initially inactive or sedentary [30]. Indeed, physical activity may induce fatigue that alters behaviour outside of physical education sessions. These physical activity consequences may persist eight months [31]. Fatigue may be associated with a number of psychological, social, and disease-related factors [32] which partly explain these results.

**Figure 4:**
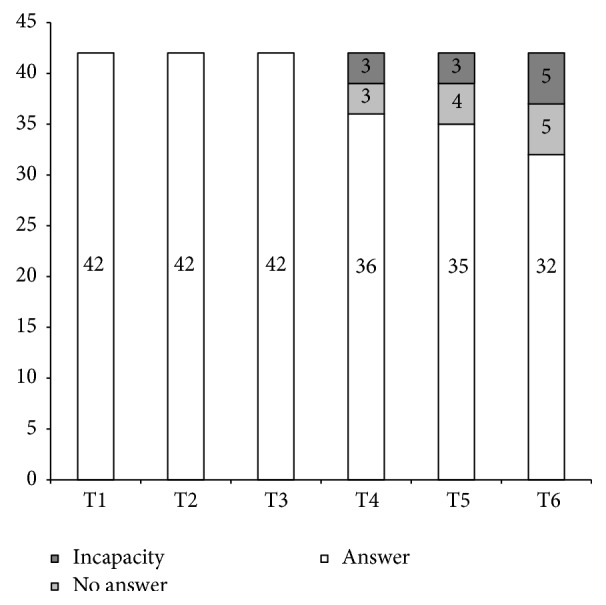
Number of participants responding, or not, to the questionnaires from baseline (T1) to 1 year after the thermal treatment (T6).

*Limitation*. This study presents several limitations. Firstly, the sample size was relatively small. We did not have adequate power to generalize the results of our study to other persons and the sample size decreased during the follow-up (Figure 3), even though >75% of the participants completed the whole protocol (Figure 4). Secondly, there is no control group. Thirdly, the evaluation of the volume of physical activity was self-reported, which may induce potential limitations (i.e., recall bias, socially desirable responses, and the influence of mood, depression, anxiety, cognition, and disability on responses) [33]. Each limitation was linked to the prospective nature of the self-controlled follow-up pilot study that can be easily offset by setting up a large-scale study (i.e., increase the sample size, use a control group, and use objective measures of physical activity).

## 5. Conclusion

One year after a 3-week additional physical education session combined with the standard thermal care, 64% of the participants included in this pilot study exhibited a higher volume of physical activity than at baseline. This volume of physical activity was, for most participants, significantly higher at each time measurement than at baseline. Moreover, the physical and psychological components of health-related quality of life changed during the follow-up (e.g., indicating a better perception of a good health during the follow-up than at baseline evaluation). Although the context of a pilot study limits the scope of our findings, this work demonstrates the feasibility of a study conducted to maintain physical activity engagement after a thermal treatment.

## Figures and Tables

**Table 1:** Anthropometric characteristics and physical fitness components before and after the period of thermal treatment and physical education sessions. Data are presented in median (minimum and maximum).

	T1	T2
BMI, kg/m^2^	28.0 (14.7–39.0)	27.6 (14.7–38.9)^*∗*^
Flexibility of the upper limb, cm	24.5 (0.0–50.0)	18.0 (0.0–52.0)^*∗∗∗*^
Flexibility of the lower limb, cm	−3.2 (−35.0–14.2)	2.7 (−26.0–18.7)^*∗∗∗*^
Heart rate, beats/min (6 MWT)	107 (82–129)	110 (80–131)^*∗∗∗*^
Maximal distance, m (6 MWT)	460 (300–604)	540 (440–665)^*∗∗∗*^
VO_2_max, L·min^−1^ (6 MWT)	2.51 (1.22–5.15)	2.74 (1.84–5.33)^*∗∗∗*^

*Note*. T1, baseline evaluation; T2, final thermal treatment evaluation. BMI: body mass index.

*∗* denotes a significant difference between T1 and T2, *p* < 0.05; *∗∗∗* denotes a significant difference between T1 and T2, *p* < 0.001.

**Table 2:** Psychological parameters at baseline and follow-up. Data are presented in median (minimum and maximum).

	T1	T2
*ISP-25*		
Global self-esteem	3.8 (2.4–5.2)	4.0 (2.6–6.0)^*∗*^
Physical self-worth	3.0 (1.0–5.0)	3.1 (1.6–5.8)^*∗∗*^
Endurance	2.0 (1.0–3.4)	2.2 (1.0–3.4)
Sport competence	1.5 (1.0–3.5)	1.5 (1.0–3.5)
Attractive body	3.7 (2.0–5.3)	3.7 (1.3–6.0)
Physical strength	1.8 (1.0–4.0)	2.0 (1.0–4.3)
*BREQ-2*		
Amotivation	0.0 (0.0–3.0)	0.0 (0.0–2.3)
External regulation	0.0 (0.0–2.5)	0.0 (0.0–3.5)
Introjected regulation	1.3 (0.0–4.0)	1.0 (0.0–3.3)
Identified regulation	2.9 (0.3–4.0)	3.0 (0.8–4.0)
Intrinsic regulation	3.0 (0.0–4.0)	3.5 (0.8–4.0)^*∗∗∗*^
*POMS-f*		
Tension-anxiety	10.0 (1.0–27.0)	4.0 (0.0–30.0)^*∗∗∗*^
Anger-hostility	8.0 (0.0–32.0)	4.0 (0.0–30.0)^*∗∗*^
Confusion-bewilderment	7.0 (0.0–13.0)	3.0 (1.0–17.0)^*∗∗∗*^
Depression-dejection	5.5 (0.0–27.0)	1.0 (0.0–50.0)^*∗∗∗*^
Fatigue-inertia	6.0 (0.0–18.0)	4.0 (0.0–19.0)^*∗*^
Vigour-activity	14.0 (1.0–25.0)	17.0 (3.0–24.0)^*∗∗*^
Friendliness	17.0 (7.0–25.0)	19.0 (8.0–25.0)
Global score	24.0 (−12.0–104.0)	2.0 (−14.0–138.0)^*∗∗∗*^

*Note*. T1, baseline evaluation; T2, final thermal treatment evaluation; ISP, Physical Self-Perception Profile (French version); BREQ, Behavioural Regulation In Exercise Questionnaire (French version); POMS, Profile of Mood States (French version).

*∗* denotes a significant difference between T1 and T2, *p* < 0.05; *∗∗* denotes a significant difference between T1 and T2, *p* < 0.01; *∗∗∗* denotes a significant difference between T1 and T2, *p* < 0.001.
